# The Effects of *Morinda citrifolia* (Noni) on the Cellular Viability and Osteogenesis of Stem Cell Spheroids

**DOI:** 10.3390/medicina56080389

**Published:** 2020-08-05

**Authors:** Sae Kyung Min, Jaekwen Oh, Jun-Beom Park

**Affiliations:** 1 Department of Periodontics, College of Medicine, The Catholic University of Korea, Seoul 06591, Korea; msek1004@naver.com; 2 Merden Dental Hospital, Bucheon-si 14544, Korea; ohjaekuen@hotmail.com

**Keywords:** cell survival, herbal medicine, Morinda, medical plants, stem cells, cell differentiation, osteogenesis

## Abstract

*Background and objectives: Morinda citrifolia* (Noni) has been widely used in herbal remedies to treat and prevent various kinds of diseases. We conducted this study to evaluate the effects of Noni extract on the maintenance of morphology, the improvement of cellular viability, and the enhancement of osteogenesis of stem cell spheroids. *Materials and Methods:* We cultured stem cell spheroids made with gingiva-derived stem cells in the presence of Noni extract at concentrations of 10, 100 and 200 ng/mL. We performed analysis of the cell morphology and changes in the cellular viability. We conducted alkaline phosphatase activity assays using a kit, and mineralization assays using an anthraquinone dye to evaluate the osteogenesis of stem cell spheroids with the addition of Noni extract. *Results:* The applied cells formed spheroids well, and the addition of Noni at 10, 100 and 200 ng/mL concentrations did not produce significant morphological changes. The quantitative values for cellular viability on Day 3 showed that the absorbance values at 450 nm were 0.314 ± 0.013, 0.318 ± 0.008, 0.304 ± 0.000 and 0.300 ± 0.011 for Noni at 0, 10, 100 and 200 ng/mL concentrations, respectively. The results of alkaline phosphatase activity with absorbance values at 405 nm were 0.189 ± 0.019, 0.174 ± 0.023, 0.192 ± 0.014 and 0.210 ± 0.062 for Noni at 0, 10, 100 and 200 ng/mL concentrations, respectively, on Day 4. There were significantly higher values of Alizarin Red S staining for Noni in the 10, 100 and 200 ng/mL groups, with the highest value at 100 ng/mL when compared with the unloaded control on Day 14. *Conclusions:* Based on these findings, we concluded that Noni extract might be applied for the enhanced osteogenic differentiation of stem cell spheroids.

## 1. Introduction

A three-dimensional culture makes cells gather and grow three dimensionally, and this creates the environment in which the cells grow and interact with the surroundings, which is more similar to in vivo situations [1,2]. It is also suggested that the gene expressions of the three-dimensional culture system simulate the gene expression in vivo, when compared with a two-dimensional culture [2,3]. Three-dimensional culture can be divided into methods with scaffold (scaffold-based system) or without scaffold (scaffold-free system) [4]. Two of the main scaffolds used for three-dimensional cultures are hydrogel and polymer [5]. However, the limitations for scaffold-based systems include a greater immune response and the limitation of the loadable number of cells [6].

Recently, scaffold-free techniques have been of great interest because this technique allows the various types of cells, including stem cells, to form cell aggregates called spheroids [6,7]. Spheroids can be fabricated in various sizes and shapes, and the reproducible size/shape of spheroids can be obtained [8,9]. Spheroids can secrete growth factors and display differential nutrient and gas availability [10]. Spheroids have been suggested as a cell delivery method for cell therapy using stem cells [11]. *Morinda citrifolia* (Noni) has been used widely in herbal remedies to treat and prevent various kinds of diseases [12]. Noni is reported to be rich in bioactive substances with significant immunomodulatory and pro-oxidant effects [13]. Noni is shown to be beneficial for the treatment of diabetes mellitus and obesity-related metabolic dysfunction [14]. Noni has been applied for the treatment of bone fractures to promote tissue regeneration [15,16]. Stem cells have been of great interest in various areas, including the tissue regeneration field [17]. A stem cell spheroid culture system utilizing stem cells has been used for cell therapy, and these systems were shown to have a differentiation ability [18]. It was shown that the application of herbal extracts may enhance the ostogenic differentiation of stem cell [19]. We conducted this study to evaluate the effects of Noni extract on the maintenance of morphology, the improvement of cellular viability, and the enhancement of the osteogenesis of stem cell spheroids.

## 2. Materials and Methods

### 2.1. Preparation of the Noni Extract

Noni was obtained from Ngaraard (Ngiwal, Palau). The age of the Noni plant was more than 15 years. The Noni fruit was ripened for 11 to 12 weeks at room temperature (20 to 28 °C), with a humidity of 60–85%. The volume and weight of the ripened Noni were 11.84 L and 18.5 kg. The final volume and weight of the Noni liquid were 10.82 L and 11 kg. Five hundred milliliters (0.508 kg) of Noni liquid was then freeze-dried in a lyophilizer (Ilshin Lab Co. Ltd., Seoul, Korea) to obtain 30 g of solid residue, producing a yield of 5.9% (w/w). Sterilized powder was mixed with distilled water and then the liquid was filtered through a 0.22 μm syringe filter.

### 2.2. Study Design and Evaluation of Spheroid Morphology

**Figure 1:**
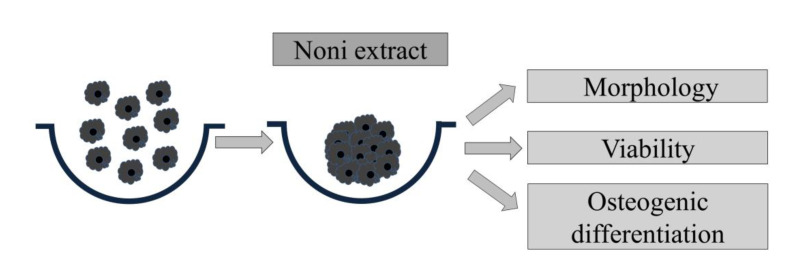
Schematic overview of the present study’s design.

Healthy gingival tissues were obtained from healthy participant undergoing periodontal surgery, as previously reported [20]. The Institutional Review Board of Seoul St. Mary’s Hospital (Seoul, Korea) (KC19SESI0280) reviewed and accredited this study. Commercially available concave microwells (H389600, StemFIT 3D; MicroFIT, Seongnam, Korea) were used to fabricate stem cell spheroids. We loaded a total of 1 × 10^6^ gingiva-derived stem cells (GMSCs) in each well and evaluated the cell response in α-minimum essential medium (MEM; Gibco; Thermo Fisher Scientific, Inc. Waltham, MA, USA) containing 15% fetal bovine serum (Gibco). After three days, cell spheroids made of GMSCs were treated with the Noni at final concentrations that ranged from 10–200 ng/mL (0 (untreated control), 10, 100 and 200 ng/mL, respectively). The morphological characteristics were evaluated on Days 1, 3, 5 and 7. Figure 1 shows an overview of the study’s design.

### 2.3. Determination of Qualitative Cellular Viability

Stem cell spheroids were cultured in growth media with the main component of α-MEM (Gibco). The commercially available two-color assay, which was based on plasma membrane integrity and esterase activity (Live/Dead Kit assay, Molecular Probes, Eugene, OR, USA), was used for the qualitative analyses of cellular viability on Days 1, 3, 5 and 7 [17]. The spheroids were treated with 2 µL of 50 mM calcein acetoxymethyl ester and 4 µL of 2 mM ethidium homodimer-1. The spheroids were incubated at room temperature for 45 min and then the spheroids were washed with the growth media. Subsequently, stem cell spheroids were observed using a fluorescence microscope at ×200 magnification (Axiovert 200; Zeiss AG).

### 2.4. Evaluation of Quantitative Cellular Viability

Quantitative cellular viability tests were performed using an assay kit based on water-soluble tetrazolium salt (Cell Counting Kit-8, Dojindo, Tokyo, Japan) on Days 1, 3, 5 and 7 [21]. Water-soluble tetrazolium salt was added to the cultures, and the spheroids were incubated for 1 h at 37 °C. Viable cells were characterized by the assay, which is based on the ability of mitochondrial dehydrogenases to oxidize water-soluble tetrazolium-8 into a formazan product. The spectrophotometric absorbance was measured with a microplate reader (BioTek, Winooski, VT, USA). Three experimental repeats were evaluated for the analysis.

### 2.5. Level of Alkaline Phosphatase Activity and Calcium Deposition

The level of alkaline phosphatase activity and an anthraquinone dye assay for calcium deposit evaluation were used to assess osteogenic differentiation [7]. Cell spheroids grown on culture plates with osteogenic media were obtained on Days 4, 7 and 14. Commercially available kits (K412-500, BioVision, Inc., Milpitas, CA, USA) were used to evaluate the level of alkaline phosphatase activity. The absorbance at 405 nm was measured after mixing a 5 mM p-nitrophenylphosphate substrate with cell lysates using an assay buffer (K412-500; BioVision, Inc.) and incubating it at 4 °C for 30 min [21].

An anthraquinone dye assay were used for calcium deposit evaluation to assess osteogenic differentiation on Days 4, 7 and 14 [22]. Spheroids were washed thrice with phosphate-buffered saline and then cell spheroids were fixed in 4% paraformaldehyde in phosphate-buffered saline at room temperature for 15 min. After that, the fixative was carefully removed and spheroids were washed three times with deionized water. Two percent Alizarin Red S Staining solution was added, and then the spheroids were incubated for 30 min. The dye was removed and the spheroids were washed three times with deionized water. The quantification of the bound dyes was performed afterwards by adding 10% cetylpyridinium chloride for 15 min at ambient temperature, and we performed spectrophotometric quantification at 560 nm [9].

### 2.6. Statistical Analysis

The results were presented as means ± standard deviations of the experiments. The experiments were performed in triplicate and tests of normality and equality of variances were performed. The Shapiro–Wilk test was used to test for normality. The differences among groups were tested by applying one-way analysis of variance with Tukey’s post hoc test (*p <* 0.05).

## 3. Results

### 3.1. Evaluation of Spheroid Morphology and Cellular Viability

**Figure 2:**
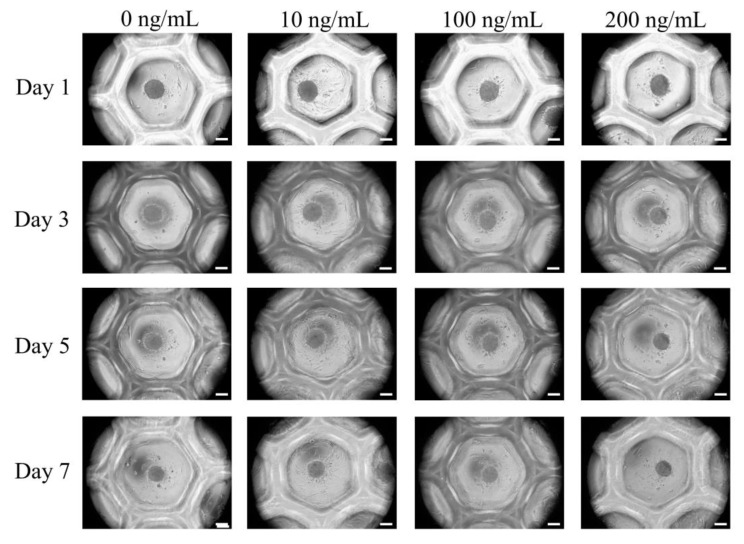
Cell morphology on Days 3, 5 and 7. The scale bar represents 100 μm (original magnification ×200).

**Figure 3:**
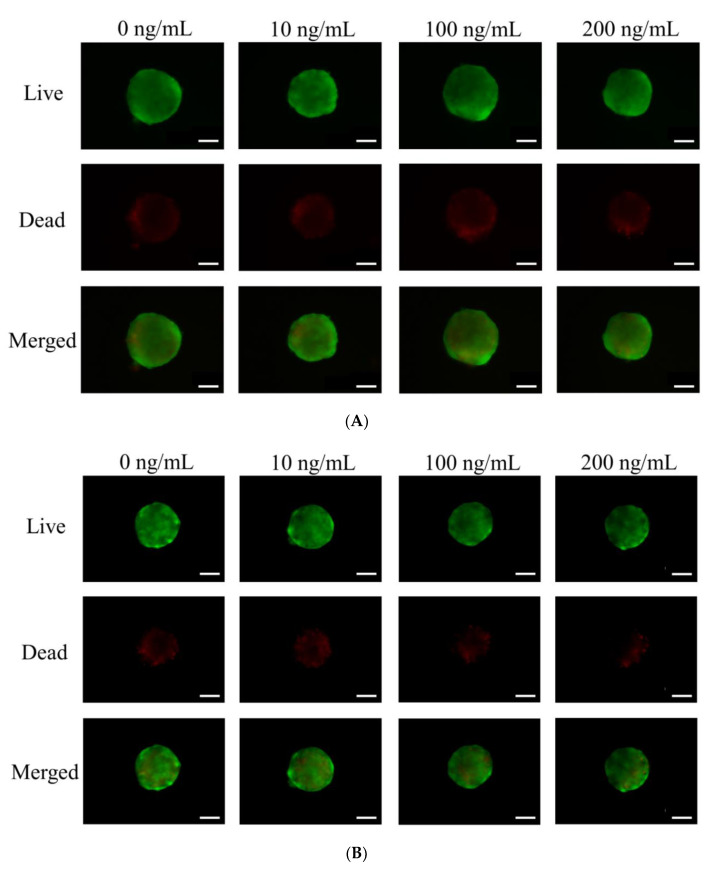

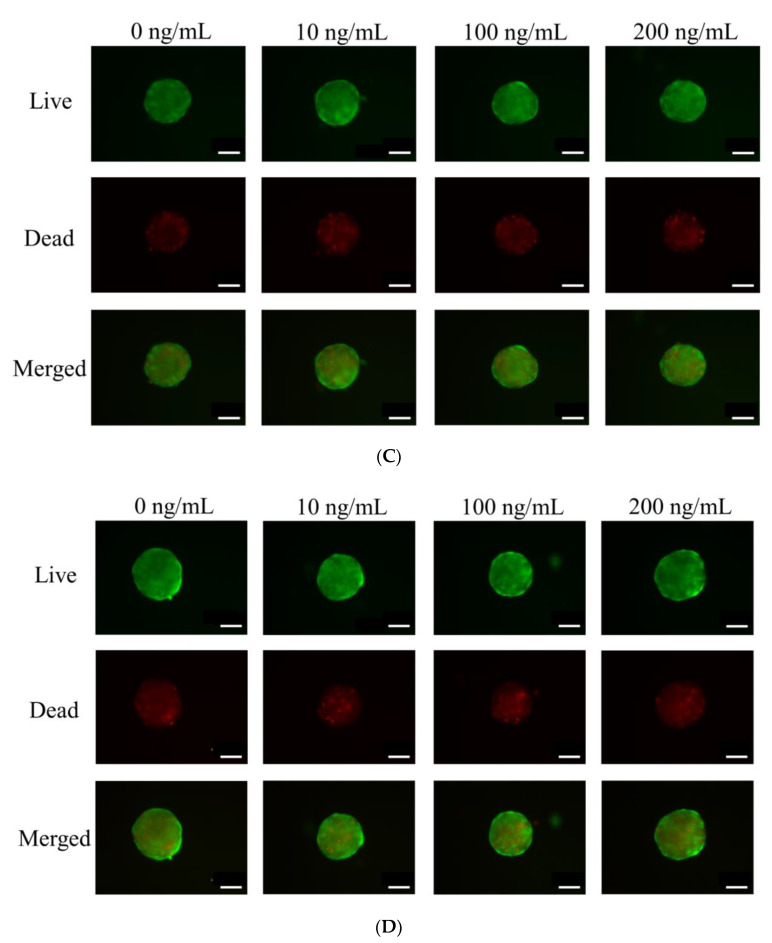
(**A**) Live, dead, and merged images of stem cells of Day 1. The scale bar represents 100 μm (original magnification ×200). (**B**) Live, dead, and merged images of stem cells of Day 3. The scale bar shows 100 μm (original magnification ×200). (**C**) Live, dead, and merged images of stem cells of Day 5. The scale bar represents 100 μm (original magnification ×200). (**D**) Live, dead, and merged images of stem cells of Day 7. The scale bar shows 100 μm (original magnification ×200).

The applied cells formed spheroids very well (Figure 2). The addition of Noni at 0, 10, 100 and 200 ng/mL concentrations did not produce significant morphological changes at Day 1. The longer incubations of Days 3, 5 and 7 did not produce noticeable changes in the shapes and sizes of spheroids. We analyzed the qualitative results on the viability of stem cell spheroids using a Live/Dead Kit assay on Days 3, 5 and 7, as shown in Figure 3. In all cases, we noticed that most of the stem cells presented green fluorescence, indicating live cells, on Day 1 (Figure 3A). We did not see any noticeable changes with longer incubation times (Figure 3B–D).

**Figure 4:**
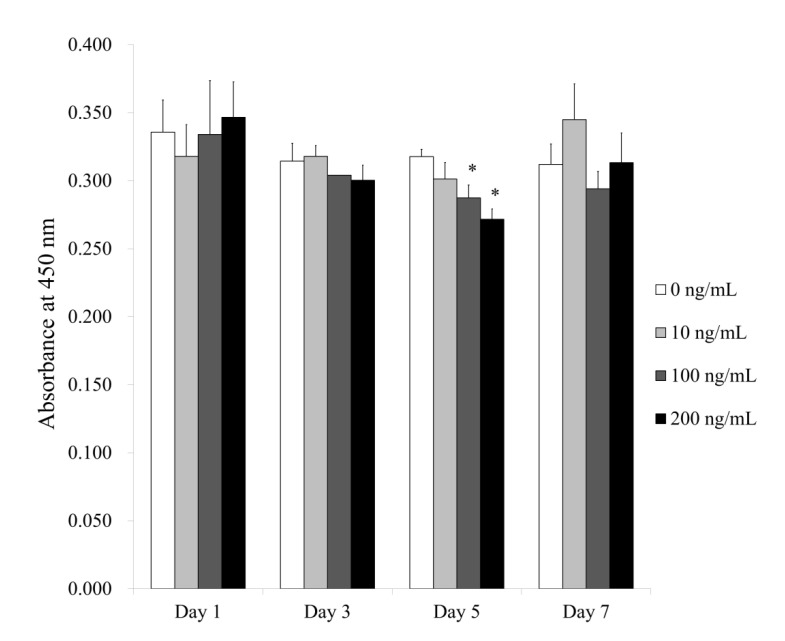
Results of quantitative cellular viability on Days 3, 5 and 7. * Statistical significances were noted when compared with the 0 ng/mL group on Day 5 (*p <* 0.05).

Figure 4 shows the quantitative values for cellular viability on Days 1, 3, 5 and 7. The absorbance values at 450 nm for Noni at 0, 10, 100 and 200 ng/mL concentrations on Day 1 were 0.336 ± 0.024, 0.318 ± 0.023, 0.334 ± 0.040 and 0.347 ± 0.026, respectively (*p >* 0.05). The absorbance values at 450 nm for Noni at 0, 10, 100 and 200 ng/mL concentrations on Day 3 were 0.314 ± 0.013, 0.318 ± 0.008, 0.304 ± 0.000 and 0.300 ± 0.011, respectively (*p >* 0.05). The absorbance values for Noni at 0, 10, 100 and 200 ng/mL concentrations on Day 5 were 0.318 ± 0.006, 0.301 ± 0.012, 0.287 ± 0.010 and 0.272 ± 0.008, respectively (*p <* 0.05). There were significantly lower values for Noni in the 100 and 200 ng/mL groups, with the lowest value in 200 ng/mL group, when compared with the unloaded control on Day 5. The absorbance values for Noni at 0, 10, 100 and 200 ng/mL concentrations on Day 7 were 0.312 ± 0.015, 0.345 ± 0.026, 0.294 ± 0.013 and 0.313 ± 0.022, respectively (*p <* 0.05).

### 3.2. Alkaline Phosphatase Activity Assays under Osteogenic Media

**Figure 5:**
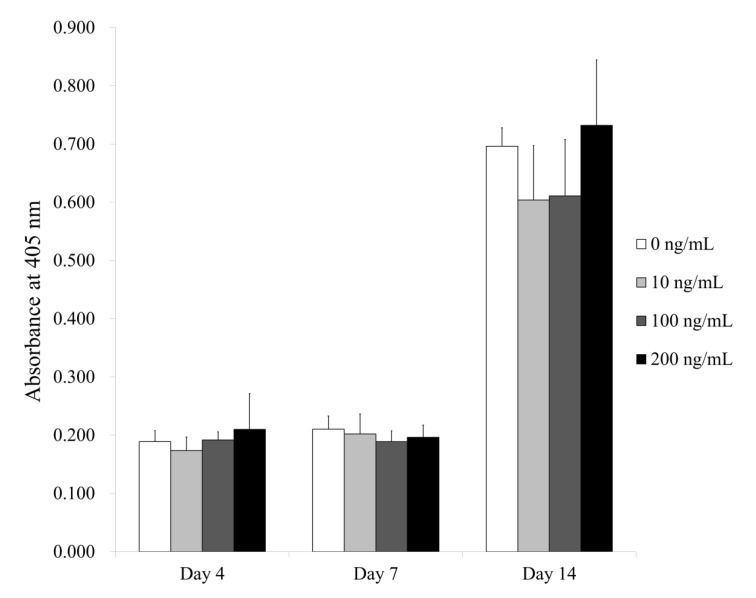
Alkaline phosphatase activity on Days 4, 7 and 14.

Figure 5 shows the results for alkaline phosphatase activity. The absorbance values at 405 nm on Day 4 for Noni at 0, 10, 100 and 200 ng/mL concentrations were 0.189 ± 0.019, 0.174 ± 0.023, 0.192 ± 0.014 and 0.210 ± 0.062, respectively (*p >* 0.05). The absorbance values at 405 nm on Day 7 for Noni at 0, 10, 100 and 200 ng/mL concentrations were 0.210 ± 0.023, 0.202 ± 0.034, 0.189 ± 0.019 and 0.196 ± 0.021, respectively (*p >* 0.05). The results on Day 14 for Noni at 0, 10, 100 and 200 ng/mL concentrations were 0.696 ± 0.032, 0.604 ± 0.094, 0.611 ± 0.097 and 0.732 ± 0.113, respectively (*p >* 0.05).

### 3.3. Evaluation of Calcium Deposition under Osteogenic Media

**Figure 6:**
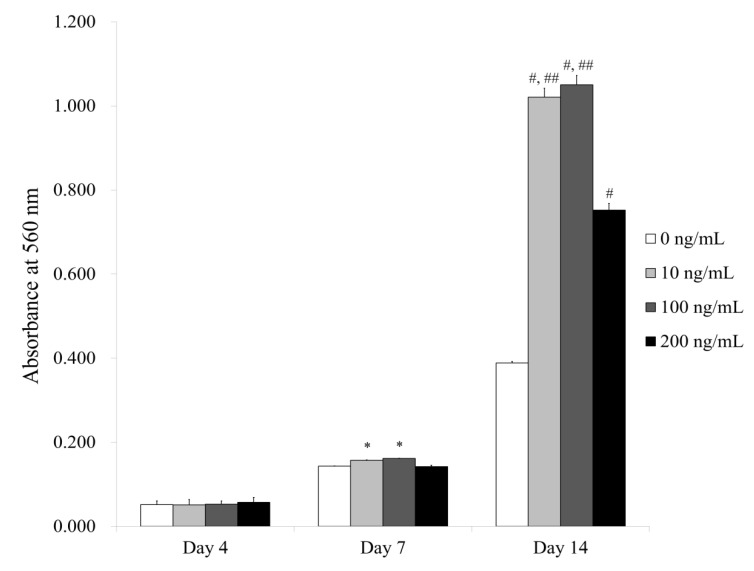
Quantitative analysis of calcium deposition on Days 4, 7 and 14. * Significant differences were noted when compared with the 0 ng/mL group on Day 7 (*p <* 0.05). ^#^ Significant differences were seen when compared with the 0 ng/mL group on Day 14 (*p <* 0.05). ^##^ Significant differences were seen when compared with the 200 ng/mL group on Day 14 (*p <* 0.05).

Figure 6 shows the quantitative results for Alizarin Red S staining. The absorbance values at 560 nm on Day 4 for Noni at 0, 10, 100 and 200 ng/mL concentrations were 0.051 ± 0.009, 0.051 ± 0.013, 0.053 ± 0.008 and 0.057 ± 0.012, respectively (*p >* 0.05). The absorbance values at 560 nm on Day 7 for Noni at 0, 10, 100 and 200 ng/mL concentrations were 0.143 ± 0.001, 0.157 ± 0.001, 0.162 ± 0.001 and 0.142 ± 0.003, respectively (*p <* 0.05). There were significantly higher values for Noni in the 10 and 100 ng/mL groups, with the highest value at 100 ng/mL, when compared with the unloaded control on Day 7. The absorbance values at 560 nm on Day 14 for Noni at 0, 10, 100 and 200 ng/mL concentrations were 0.388 ± 0.004, 1.021 ± 0.021, 1.050 ± 0.023 and 0.752 ± 0.016, respectively (*p <* 0.05). There were significantly higher values for Noni in the 10, 100 and 200 ng/mL groups, with the highest value at 100 ng/mL, when compared with the unloaded control on Day 14.

## 4. Discussion

This study evaluated the effects of Noni extract on the maintenance of morphology, the improvement of cellular viability, and the enhancement of the osteogenic differentiation of stem cell spheroids.

Noni is a tropical tree with an ovoid yellow fruit, and is distributed widely in areas of Southeast Asia, Australia, Hawaii, Tahiti and Micronesia [23]. The Noni fruit is consumed as food and has been approved as a commercial food [24]. Noni was reported to contain broad therapeutic properties, especially in the medicinal field [25]. A more recent report showed that Noni had antioxidant and antibiotic characteristics in vitro [26]. Noni has been used to treat inflammatory diseases, atopic dermatitis, colitis, cervical spondylosis, hypertension and cancer [27,28]. A previous report showed that Noni fruit extract had potent inhibitory activity on gut bacterial β-glucuronidase [29]. Noni extract has been shown to have antibacterial activity against *L. monocytogenes* [30]. Another in vitro study showed that Noni showed antimicrobial activity against *Enterococcus faecalis* and *Candida albicans* [31]. In this study, the application of Noni produced increased osteogenic differentiation.

Various compounds identified from Noni include flavonoids, anthraquinones, glycosides, alkaloids, carotene terpenoids, vitamin A and vitamin C [14,26]. Polysaccharides from Noni have been shown to modulate regulatory process of the cells and to be involved in immunomodulation [13]. Polysaccharides from Noni have been shown to possess anti-inflammatory functions [32]. Damnacanthal, which is a type of anthraquinone from Noni, is reported to have anticancer properties [26].

Various methods have been used for the extraction of Noni, which includes hot water extraction, methanolic extraction, ethanol extraction, ultrasonic-assisted extraction, and pulsed electric field-assisted extraction [25,33,34]. It has been shown that the extraction yields, sugar contents, monosaccharide proportions, uronic acid contents, antioxidant activities and antiproliferative abilities differed between the groups [33].

Various doses have been used for testing the effects of Noni [25,28,35]. The toxicological studies revealed that higher doses of Noni fruit juice of 4000 mg/kg or 5000 mg/kg for two or more months may cause toxic effects on the liver and kidneys [28]. Doses of 500–1000 mg/kg of Noni extract produced heptoprotective effects, proven by an increase in the liver antioxidant enzyme and alkaline phosphatase activity [25]. For in vitro experiments, 0.4, 1.2, 3.7, 11.1, 33.3 and 100 µg/mL concentrations were used to perform cell viability assays [35]. Researchers have applied the concentrations of 25, 50 and 100 µg/mL for the cell colony-formation assays [35]. In this study, we treated cell spheroids made of GMSCs in the presence of the Noni at final concentrations that ranged from 10 to 200 ng/mL (0 (untreated control), 10, 100 and 200 ng/mL). There was significantly enhanced osteogenic differentiation for Noni in the 10, 100 and 200 ng/mL groups, with the highest value at 100 ng/mL, when compared with the unloaded control.

## 5. Conclusions

Based on these findings, it can be suggested that Noni extract might be applied for the enhanced osteogenic differentiation of stem cell spheroids.
